# Supplementary material for: The variability of inner ear orientation in saurischian dinosaurs: testing the use of semicircular canals as a reference system for comparative anatomy
Source: PeerJ. 2013 Aug 6;1:e124. doi: 10.7717/peerj.124 (PMC3740149; doi:10.7717/peerj.124)
Supplement: Supplemental Information 2 — Protocol for the digitalization of landmarks and Generalized Procrustes Analysis (GPA). [file peerj-01-124-s002.doc]

**SUPPLEMENTARY MATERIAL:**

**The variability of inner ear orientation in saurischian dinosaurs: Testing the use of semicircular canals as a reference system for comparative anatomy**

Jesús Marugán-Lobón^1,2^ and Luis M. Chiappe^2, *^ and Andrew A. Farke^3^

1 Unidad de Paleontología, Dpto. Biología, Universidad Autónoma de Madrid, 28049 Cantoblanco (Madrid). Spain. 2 The Dinosaur Institute, Natural History Museum of Los Angeles County, Los Angeles, CA 90007. U.S.A. 3 Raymond M. Alf Museum of Paleontology, Claremont, California USA

**Protocol for the digitalization of landmarks and Generalized Procrustes Analysis (GPA)**

This protocol is intended to help the user apply Geometric Morphometrics (GM) to orient dinosaur skulls within the comparative framework proposed in this study, using 5 landmarks in 2D. The application of GM involves a two-step procedure (A and B, below). First, it is necessary to acquire the raw data for any new specimen, that is, the digitalization of the landmark coordinates on the skulls, and second, it requires performing the Procrustes superimposition of the landmark configurations that references the data to a common set of coordinates (the sample’s Procrustes mean), taking into account the spatial directions of a crocodile relative to gravity. Here we explain this procedure in nine steps. Multiple GMM programs are freely available at <http://life.bio.sunysb.edu/morph/>. Most of the software is Windows-based, but all of them work very well with compatibility layers such as WINE (Linux), and any emulator for the Macintosh OS. In this study we provide our data in ***.tps*** file format, which is a standard in GM that most programs read. For landmark digitalization we recommend using **TPSdig2**, **TPSrelw** for superimposition both by F.J. Rohlf (v. 2010), and the program **Morpheus et al.** for visualization (both the classic version and the new version in JAVA© by D.E. Slice (2002 and 2007, respectively).

The stepwise procedure is to add (paste) any new specimen’s data (landmark coordinates) into our data set. If working with only your own data, notice that it is *necessary* to have at least three specimens (because the new coordinate system is an average). Because our GPA results are oriented to the posture of a crocodile (*C. johnstoni*), the latter always needs to be the included as the first specimen in the sample (unless you have your own crocodile data). That is, as long as the crocodile is kept as the first entry, all the Procrustes aligned specimens (i.e., referenced to the mean) are oriented with respect to the crocodile. The user can include any organism’s skull, with the only limitation that the landmarks need to be homologous (i.e., correspond to the same structure within the skull). As well, there can be **no missing landmarks**.

**IMPORTANT: To use our data, the numbering of the landmarks needs to be consistent with that used in our study.**

**A. Data acquisition and landmarks**

**1^st^ step:** Take a picture in lateral view of the skull or skulls to be coordinated. It is important to make sure that the picture correctly captures the skull in lateral view (i.e., avoiding any parallax). Here we will use images in the *.jpg* compression format, but any other type can also be used and will not affect the analysis. **Having a good quality picture is necessary to be able to see details of the anatomy and be able to place the landmarks correctly. A high resolution image is not strictly necessary and 1200x800 resolution or less should be sufficient. All skulls have to be facing the same directions.**

**2^nd^ step:** Make a text file with any text processor (e.g., Notepad or gedit), and type ‘LM=0’. Then press enter to jump to the next line, type IMAGE=*nameofyourpicture.jpg*, press enter, and finally type ID=*nameofyourspecimen*. Then save the file (see Figure below).

**3^rd^ step:** Install and start TPSdig2, and load the .tps file in order to place the landmarks. For this procedure, go to File->Input source->File, and find and load the .tps file with the information about your specimen/s. Once the image is shown, simply start placing the five landmarks with the mouse, and then save.


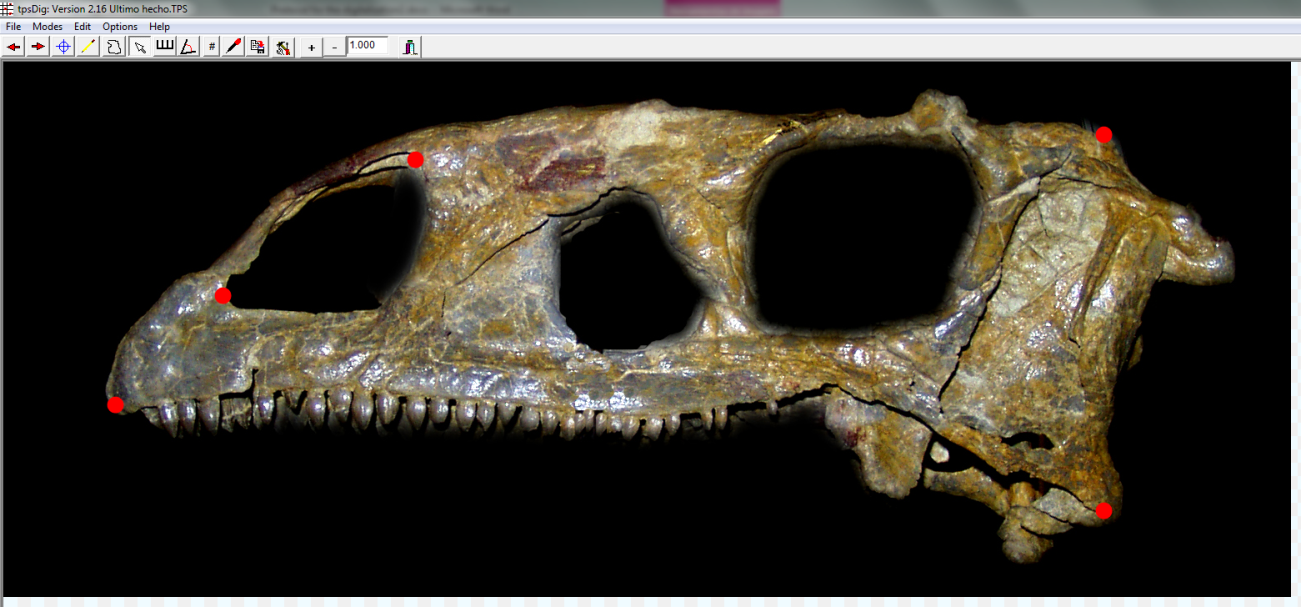


**Figure 2.** In the TPSdig2 program images are NOT loaded directly. Instead, the program reads the *.tps* file previously made, which is why it is important that the IMAGE=*picture.jpg* correctly addresses the name of the image and that both the *.tps* file and the image are put in your computer in the same folder. Notice the arbitrary position of the skull of *Plateosaurus*.

**4^th^ Step:** Open your *.tps* file as if it were a text file (i.e., with a text editor). You will see that TPSdig2 changed the command LM=0 to LM=5, and there will be a set of five landmark’s *x* and *y* coordinates below.

**5^th^ step:**  Select all this information and copy it. Then, open our *GPA Ordered Dinosaur data.tps* as if it were a text file (i.e., with a text editor) and paste your data below our last individual in the last row of the file (see Fig. 3), and save.

**Figure 3.** Position in our .tps file where data need to be pasted.

**B. Applying the Procrustes method**

**6^th^ step:** Once data are ready, the next step is to load the data into a Geometric Morphometrics program to perform the Procrustes superimposition. Several programs will do this, but we’ll explain TPSrelw. Download it from the Stony Brook server and install.

**In TPSrelw:** Load the *.tps* containing all the specimens by pressing the **DATA** button. Then, press the **CONSENSUS** button; this will apply a GPA to the raw data. Then select File->Save->Aligned specimens to save the transformed data. Any name, but make sure to save in ***.tps*** file format. This new file contains the shape data oriented relative to the Procrustes mean (Fig. 4).

**Figure 4.** TPSrel GUI (left) and GPA Superimposed specimens (right)

**7th step:** For visualization, install Morpheus et al. (old version, or v. 2007), select File->Import->tps and select your .tps file with the GPA shape data. The program will show the first specimen, so you only need to choose your specimen in the OBJ tab, found at the lower left corner of the screen (Fig. 5). In the option ‘Graphics’, it is possible to save the resulting image in.png format. Alternatively, one can simply perform a screen capture. Notice that the GPA results are referenced to the mean, but all of these data as a whole are oriented to the spatial directions of the croc.


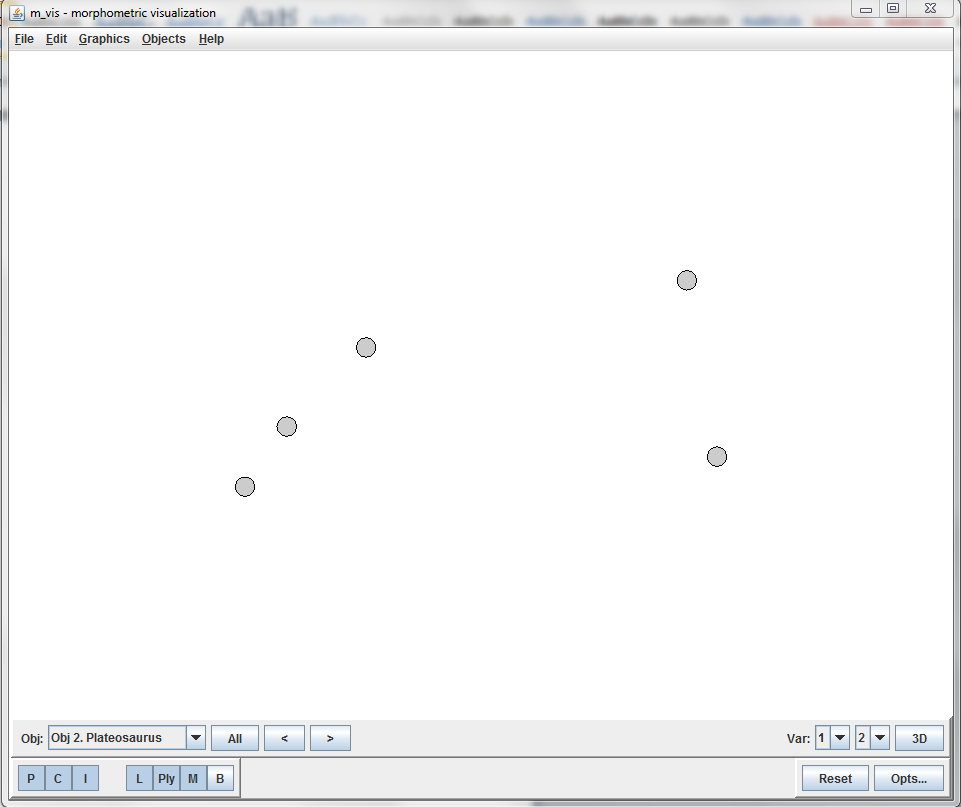


**Figure 5.** Morpheus (v.2007) GUI. Commands in the lower corner allow you to display individuals separately.


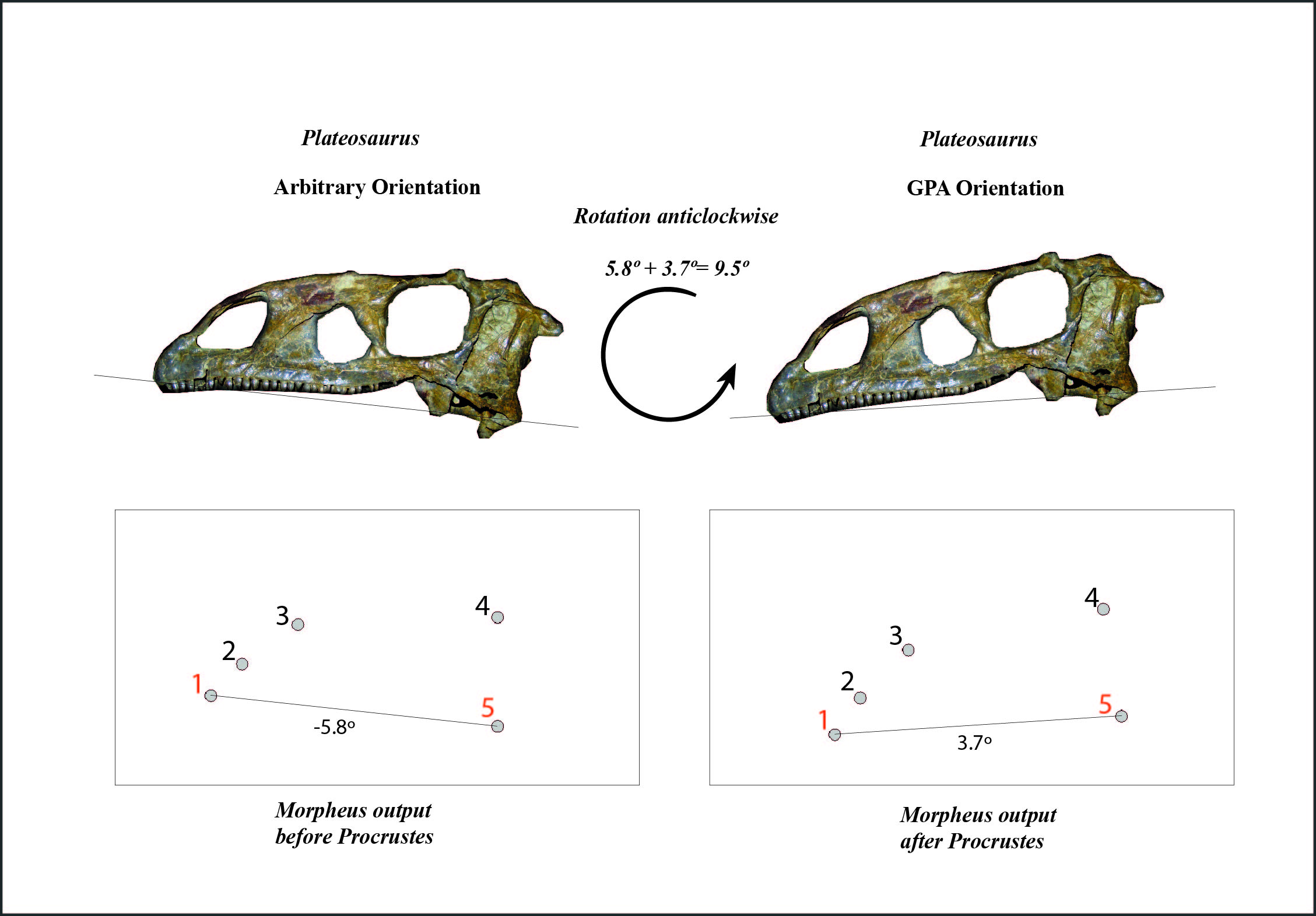
**8^th^ Step:** Reorient the image of your specimen relative to the new position acquired by GPA of the landmarks by measuring the difference between the original orientation and the resulting orientation. The easiest standard is to use two landmarks, **particularly useful are those on the rim of the facial skeleton (LM 1 and 5)**. Programs useful for this purpose include Illustrator, Corel Draw, Photoshop, GIMP, Inkscape, etc. Measurement of the angle of rotation is very simple in TPSdig2. After this rotation, your specimen is in the standardized orientation relative to the dataset.

**Figure 6.** Example of how to reorient a skull with vector graphics software (e.g. Illustrator).
